# Mortalidad evitable en la niñez en Colombia: una forma de violencia

**DOI:** 10.7705/biomedica.7910

**Published:** 2026-06-01

**Authors:** Ángela María Hoyos-Quintero, Alejandra María Díaz-Tamayo, Herney Andrés García-Perdomo

**Affiliations:** 1 Universidad del Valle, Cali, Colombia Universidad del Valle Cali Colombia; 2 Unidad de Urología-Urooncología, Departamento de Cirugía, Escuela de Medicina, Universidad del Valle, Cali, Colombia Departamento de Cirugía Escuela de Medicina Universidad del Valle Cali Colombia

**Keywords:** mortalidad infantil, mortalidad del niño, violencia, pobreza, Infant mortality, child mortality, violence, poverty

## Abstract

¿Es la mortalidad evitable en la niñez en Colombia una forma de violencia? El análisis de la mortalidad en la infancia está superando el enfoque netamente biológico, pues esta situación es reflejo de la confluencia de aspectos sociales, ambientales, económicos y políticos entre otros, los cuales retratan la vulnerabilidad de este grupo poblacional. A nivel mundial, la lucha para controlar la mortalidad ha generado grandes logros, mientras que los países de medianos y bajos ingresos aún presentan cifras altas y dispares entre regiones, relacionadas principalmente con la violencia como un fenómeno latente. Por ello, el objetivo de este ensayo es reflexionar sobre la mortalidad en la infancia como una forma de violencia indirecta.

En conclusión, la mortalidad evitable en la niñez requiere ser analizada desde una visión política que permita identificar las diferentes violencias que se retratan en este suceso. Por ello, la mortalidad evitable en la niñez como una forma de violencia requiere ser analizada desde un enfoque intersectorial y multidisciplinar que permita priorizar acciones para controlarla.

## Abstract (en)

Is avoidable childhood mortality in Colombia a type of violence? Childhood analysis mortality requires a broader approach than a purely biological one, as this situation reflects the confluence of social, environmental, economic, and political factors, among others, which portray the vulnerability of this population group. At the global level, efforts to control mortality have yielded significant achievements; however, low- and middle-income countries still show high and uneven figures across regions, related primarily to violence as a latent phenomenon.

The objective of this essay was to reflect on childhood mortality as a form of indirect violence. In conclusion, avoidable childhood mortality needs to be analyzed from a political perspective that allows for the identification of the different forms of violence portrayed in this event. Consequently, it can be stated that avoidable childhood mortality as a form of violence requires analysis from an intersectoral and multidisciplinary approach that enables the prioritization of actions to control it.

El estudio de la mortalidad en la niñez ha ocupado, en gran medida, la investigación en salud pública y se ha orientado desde la mirada biológica al indagar las causas de la morbilidad ^1^^-^^9^ y, recientemente, los enfoques han incluido una mirada social que busca la asociación entre los factores determinantes socioeconómicos y la mortalidad ^1^^,^^10^^-^^31^ y nuevas miradas integradoras convocan (sic) a la situación sociopolítica y al entorno donde ocurren diferentes tipos de violencia ^32^^,^^33^, reconociendo la importancia del entorno en la comprensión de los riesgos y vulnerabilidades de la infancia.

Para esta argumentación se convoca (sic), se explora el tema de la mortalidad en la niñez desde una visión social de la salud. Se hace énfasis en la pregunta de cómo la mortalidad en la niñez se convierte en una forma de violencia en relación con la sociedad donde ocurre, lo cual permite visibilizar la indiferencia que se ha tenido, tanto del Estado como de nosotros como sociedad, ante esta problemática.

La mortalidad infantil está condicionada por factores determinantes sociales y ambientales. Más allá de los factores biológicos, su incidencia se explica en gran medida por las desigualdades estructurales que configuran el entorno social. Aspectos como el acceso a los recursos económicos, la calidad y seguridad alimentaria, la disponibilidad de agua potable, los niveles de pobreza y el grado de escolaridad conforman un entramado de factores determinantes sociales de la salud que guardan una relación directa con la estratificación socioeconómica, y que condicionan de manera significativa las oportunidades de supervivencia en la niñez.

El análisis de la mortalidad infantil desde una perspectiva integral ha implicado un cambio en las intervenciones de salud pública. Este enfoque trasciende la visión reduccionista de carácter biológico -que atribuía la mortalidad exclusivamente a la presencia de enfermedades- para reconocer la interacción de factores determinantes sociales, económicos y ambientales que condicionan y explican la ocurrencia de este desenlace. Sin lugar a dudas, un niño que se contagie de un «resfriado» o de un virus que le afecte el sistema digestivo, tiene un riesgo diferencial de enfermar gravemente al pertenecer a una clase social baja ^28^^,^^33^.

Esta realidad ubica a los niños pobres en una situación de vulnerabilidad tanto externa como interna ^33^^,^^12^. La vulnerabilidad externa, en términos de Amartya Sen, no se limita a la falta de ingresos, sino que refleja una desventaja estructural más profunda: la privación de capacidades y la restricción de libertades fundamentales que impiden a los niños desarrollar plenamente su potencial ^34^.

Por otra parte, la vulnerabilidad interna, según Rutter ^35^, no constituye un rasgo fijo, sino una susceptibilidad a sufrir efectos negativos frente a situaciones adversas como la pobreza, la violencia o el abandono, que resulta de la interacción entre predisposiciones biológicas y condiciones de vida. Esta circunstancia impacta directamente los procesos emocionales, cognitivos y en la salud general de los niños.

Así, la muerte de cualquier niño por enfermedades evitables expresa no solo su fragilidad biológica, sino también, la precariedad social de su entorno, marcada por la inestabilidad de ingresos monetarios, el desempleo o el trabajo informal, la ausencia de seguridad social y las limitaciones educativas que impiden reconocer y prevenir riesgos. Esta postura respalda la tesis de la presente argumentación, en la que se asume que la mortalidad en la niñez ocurre por la relación que existe entre esta y el contexto sociopolítico del entorno y cómo estos aspectos interactúan junto con enfermedades que aumentan el riesgo de fallecer.

En varios países de Latinoamérica, existen características similares que exponen a sus habitantes a altos índices de violencia, corrupción e inequidad en los ingresos y en el acceso a la educación y, por ende, la inequidad en salud ^36^. Estos aspectos reflejan el riesgo de los niños de esta región que se encuentren en situación de vulnerabilidad. Por tal razón, se plantea como objetivo reflexionar sobre la mortalidad en la infancia como una forma de violencia indirecta.

## Desarrollo

A partir de un ejercicio reflexivo y argumentativo sobre los aspectos relacionados con la mortalidad en la niñez en el proceso de diseño de una investigación doctoral, se encontraron diferentes formas de analizar esta temática: desde la visión de los factores determinantes sociales de la salud y los determinantes políticos, y desde el enfoque de violencia como retrato de la realidad de países como Colombia.

En Latinoamérica, la tasa de mortalidad en la niñez para el 2022 varió entre 6 y 24 muertes de niños menores de 5 años por cada 1000 nacidos vivos. El país con la tasa más baja fue Chile (6 muertes por cada 1000 nacidos vivos) y los de tasas más elevadas mientras que las tasas más elevadas fueron Bolivia (23 muertes por cada 1000 nacidos vivos) y Venezuela (24 muertes por cada 1000 nacidos vivos) ^37^.

En países como Colombia, donde el conflicto interno ha persistido por más de un siglo ^38^, genera efectos en las personas tanto en su salud física como mental. La muerte y la violencia forman parte de la cotidianidad, y esta realidad genera la inquietud de si los ciudadanos están habituados a la violencia y a la muerte. Esto puede ejemplificar un fenómeno de «normalización de la violencia» ^38^, de costumbre, al ser lo cotidiano vivir en medio de la guerra ^39^, o todo pueda resumirse en la palabra «resiliencia».

La mortalidad en la niñez en países como Colombia ocurre como una interacción de diferentes aspectos que se relacionan entre sí, estudiados probablemente de manera aislada, pero pocas veces analizados desde una visión integradora y compleja ^22^^,^^40^^-^^42^. Este problema de la mortalidad en la niñez va más allá de analizar la interrelación entre aspectos como la enfermedad, el nivel de vida y los factores determinantes socioeconómicos e, incluso, con los aspectos sociales. Todo este análisis quedaría incompleto sin tener en cuenta el contexto en el que ocurre y la presencia de un conflicto permanente como el que se vive en el país.

Para facilitar la comprensión de esta realidad, se considera lo propuesto por Galtung, quien plantea el «triángulo de la violencia» compuesto por la violencia directa, estructural y cultural ^43^. Para este autor, la violencia directa es la manifiesta, evidente y palpable; mientras que la violencia estructural o indirecta es intrínseca a los sistemas sociales, políticos y económicos que gobiernan las sociedades, los estados y el mundo; y la violencia cultural se asume como aquellos aspectos de la cultura y de la simbología que puedan justificar la violencia directa ^44^.

Aunque la sociedad colombiana se ha habituado a la violencia directa ^45^, haciendo cotidianos los niveles de agresión ante la presencia de un conflicto así sea de nivel bajo, como los relacionados con la convivencia, existe una forma de violencia que es difícil visualizar y que está compuesta por la vulneración de los derechos de los individuos por bloqueo o limitación de acceso, como a nutrición adecuada, educación, atención médica brindada de manera diferencial según las características de la población y el entorno en el que viven, entre otros. Esto termina siendo fatal en el caso de los grupos vulnerables como los niños y, más aún, si se encuentran en situación de pobreza. Esta realidad ejemplifica la violencia estructural o indirecta a la que están expuestas las personas en el país.

Teniendo en cuenta estos aspectos, la mortalidad en la niñez se configura como una forma de violencia estructural, ejemplificándose en el análisis de los aspectos que la determinan: acceso diferencial a los servicios de salud según el nivel económico de la familia, el sistema de protección social en salud, condiciones de violencia social y demás aspectos que según la literatura aumentan el riesgo de fallecer en condiciones de pobreza y vulnerabilidad. Esto se configura como violencia indirecta, invisible y oculta tras las otras formas de analizarla.

Aunque en Colombia se han conformado equipos profesionales entre las secretarías de salud departamentales, las secretarías de salud municipales, las instituciones prestadoras de servicios de salud y, en algunos casos, representantes de la comunidad, con el fin de realizar una reunión, llamada «unidad de análisis» en la que se estudia y discute la forma en que ocurrió la muerte infantil y los sucesos que pudieron aportar para su ocurrencia ^46^, lo que permitiría escuchar varios enfoques, unos relacionados con la explicación biológica y otros con la decisión de cuidado que toman tanto la familia y la madre como la institución, en la mayoría de estas reuniones no se escuchan las voces de las familias y de las madres, lo que limita el análisis a los profesionales de salud y genera una visión reduccionista de las formas de violencia, que evitan su identificación.

Por ello, las explicaciones que se relacionan con la demora en la atención, en buscar asistencia médica, dificultades de acceso a los servicios de salud, y la «negligencia de las madres» son las más frecuentemente encontradas ^47^. A pesar de que, en algunos casos, la relación entre el tiempo de aparición de los síntomas y el desenlace fue largo (meses), en otros casos ocurría en días. La constante en los análisis es el conocimiento y las actitudes de prácticas en salud relacionadas con las madres y las familias; sin embargo, este aspecto no permite evaluar o conocer el nivel educativo de la madre o tener conocimiento directo de lo que conocía.

No obstante, los análisis se realizan de manera rigurosa, pero con datos incompletos. En muchas ocasiones, cada palabra queda consignada en el papel, pero las acciones que se generan a nivel regional para evitar otras muertes con características similares no alcanzan a controlar la evolución de otros sucesos.

Aunque en los estudios se menciona el nexo entre pobreza y mortalidad en la niñez ^2^^,^^17^^,^^18^^,^^23^^,^^24^^,^^34^^,^^48^^,^^49^, convirtiéndose en una explicación obvia, no deja de asombrar que, aun siendo obvio, no lo sea para el Estado. Si bien esto es cierto, la pobreza es multifactorial, por lo que las políticas públicas deberían tener transversalidad, siendo planteadas en conjunto, pues la salud de la población depende de factores tanto biológicos como sociales, económicos, políticos y espirituales ^46^^,^^50^^-^^55^. No sería difícil determinar un responsable de «no hacer», pero ese «no hacer» tiene múltiples responsables y puede, sin lugar a duda, generar conflictos que la sociedad no está preparada para asumir.

De esta manera, es más fácil explicar la mortalidad en la niñez a partir de su relación con enfermedades comunes en la infancia que pueden aumentar su efecto en condiciones de malnutrición y pobreza, así si el Estado decide implementar un programa de alimentación temporal e interviene a las familias, la solución será fácil, así sea temporal.

Es por esta razón que la mortalidad en la niñez sigue siendo alta en los países en vías de desarrollo, porque nadie asume la responsabilidad y todos se conforman con el paño de agua tibia en la frente de un enfermo con fiebre crónica.

## Consideraciones finales

La visión política de la mortalidad evitable en la niñez en países con antecedentes de violencia permitiría visibilizar realidades hasta ahora ocultas, identificar factores que faciliten dicho análisis y priorizar acciones dirigidas al control de la mortalidad de los niños en las regiones. Por esta razón, la salud en la niñez y sus afectaciones requieren un enfoque transectorial y multidisciplinar que permita a las instituciones tener insumos para la formulación de acciones en salud que propendan por el mantenimiento de la salud de la infancia.
